# Aberrant impulse control circuitry in obesity

**DOI:** 10.1038/s41380-022-01640-5

**Published:** 2022-06-14

**Authors:** Daniel A. N. Barbosa, Fiene Marie Kuijper, Jeffrey Duda, Allan R. Wang, Samuel C. D. Cartmell, Sabir Saluja, Tricia Cunningham, Rajat S. Shivacharan, Mahendra T. Bhati, Debra L. Safer, James D. Lock, Robert C. Malenka, Ricardo de Oliveira-Souza, Nolan R. Williams, Murray Grossman, James C. Gee, Jennifer A. McNab, Cara Bohon, Casey H. Halpern

**Affiliations:** 1 Department of Neurosurgery, Pennsylvania Hospital, Perelman School of Medicine, University of Pennsylvania, Philadelphia, PA USA; 2 Department of Neurosurgery, Stanford University School of Medicine, Stanford, CA USA; 3 Department of Radiology, Perelman School of Medicine, University of Pennsylvania, Philadelphia, PA USA; 4 Department of Psychiatry and Behavioral Sciences, Stanford University School of Medicine, Stanford, CA USA; 5 Nancy Pritzker Laboratory, Department of Psychiatry and Behavioral Sciences, Stanford University School of Medicine, Stanford, CA USA; 6 Department of Specialized Medicine, Gaffrée e Guinle University Hospital, The Federal University of the State of Rio de Janeiro, Rio de Janeiro, Brazil; 7 Department of Neurology, Perelman School of Medicine, University of Pennsylvania, Philadelphia, PA USA; 8 Department of Radiology, Stanford University School of Medicine, Stanford, CA USA; 9 Department of Surgery, Corporal Michael J. Crescenz Veterans Affairs Medical Center, PA Philadelphia, USA

**Keywords:** Neuroscience, Psychiatric disorders

## Abstract

The ventromedial prefrontal cortex (vmPFC) to nucleus accumbens (NAc) circuit has been implicated in impulsive reward-seeking. This disinhibition has been implicated in obesity and often manifests as binge eating, which is associated with worse treatment outcomes and comorbidities. It remains unclear whether the vmPFC-NAc circuit is perturbed in impulsive eaters with obesity. Initially, we analyzed publicly available, high-resolution, normative imaging data to localize where vmPFC structural connections converged within the NAc. These structural connections were found to converge ventromedially in the presumed NAc shell subregion. We then analyzed multimodal clinical and imaging data to test the a priori hypothesis that the vmPFC-NAc shell circuit is linked to obesity in a sample of female participants that regularly engaged in impulsive eating (i.e., binge eating). Functionally, vmPFC-NAc shell resting-state connectivity was inversely related to body mass index (BMI) and decreased in the obese state. Structurally, vmPFC-NAc shell structural connectivity and vmPFC thickness were inversely correlated with BMI; obese binge-prone participants exhibited decreased vmPFC-NAc structural connectivity and vmPFC thickness. Finally, to examine a causal link to binge eating, we directly probed this circuit in one binge-prone obese female using NAc deep brain stimulation in a first-in-human trial. Direct stimulation of the NAc shell subregion guided by local behaviorally relevant electrophysiology was associated with a decrease in number of weekly episodes of uncontrolled eating and decreased BMI. This study unraveled vmPFC-NAc shell circuit aberrations in obesity that can be modulated to restore control over eating behavior in obesity.

## Introduction

Untreated impulsivity and associated maladaptive behavior underlie a multitude of public health crises. Readily available, highly palatable and refined foods that predispose to impulsive overeating has repeatedly been linked to obesity, a condition at worldwide epidemic proportions [1]. Further, such disinhibition over how much one eats has been associated with refractoriness of obesity to even the most aggressive of treatments [2, 3]. Binge eating may be the most extreme behavioral ramification of such disinhibition given the quantity of food consumed in one episode [4, 5]. Not surprisingly, obesity is 3–6 times more common among subjects engaging in binge eating [6]. Binge eating not only exacerbates treatment outcomes for obesity, but also predisposes to impaired metabolic function, psychiatric comorbidities, and diminished quality of life, in part, due to debilitating preoccupations with food [7–9]. Thus, examining the neural underpinnings of obesity when disinhibited eating is present may elucidate the role of specific neural circuits, uncover disease mechanisms not previously understood, and guide neuromodulatory interventions tailored to these commonly coexisting conditions [1].

Nucleus accumbens (NAc) deep brain stimulation (DBS) is one neuromodulatory strategy that has exhibited promise in ameliorating disinhibited eating and inducing weight loss in mice [10, 11]. Single cases of human subjects with obesity undergoing NAc DBS and experiencing restored impulse control and substantial weight loss have also been reported [12, 13]. In mice, NAc DBS delivered in response to electrographic activity preceding hedonic feeding enhanced local calcium signaling, a surrogate for action potentials, and this effect was associated with decreased consumption of a highly palatable diet [11, 14]. It is also expected that NAc DBS effects impact circuitry beyond the NAc [15]. Of particular interest is the ventromedial prefrontal cortex (vmPFC), which has exhibited activation based on c-Fos expression and blood‐oxygenation‐level‐dependent signal across small and large animal species, respectively, during NAc DBS [16, 17]. These findings are not surprising, as the vmPFC-NAc circuit has been repeatedly implicated in disinhibition in general [17–19]. Conversely, local vmPFC activation has suppressed appetitive eating [18, 20]. Thus, the therapeutic effects of NAc DBS in collectively treating obesity and related disinhibition toward food are likely due, at least in part, to modulation of this circuit [21–23].

Here, we performed a multimodal imaging analysis to examine this putative circuit’s involvement in disinhibited eating in obesity. We (1) analyzed normative diffusion MRI data from the Human Connectome Project (HCP) to localize where vmPFC streamlines converge within the NAc. To examine the relevance of this circuit in the context of obesity, we then (2) applied the NAc subregion where vmPFC streamlines converge to a cohort of females with and without obesity that engaged in binge eating (i.e., binge-prone cohort), and investigated the functional and structural connectivity of this circuit, as well as vmPFC thickness, in relation to the obese state in the binge-prone cohort. Finally, we (3) directly probed this circuit in one binge-prone, obese female who received closed-loop DBS encompassing the NAc as part of a first-in-human, interventional study [24].

## Materials and methods

### Imaging data

MRI acquisition parameters are summarized in Supplementary Table 1. We included MRI data from three different cohorts: (1) publicly available, high-resolution, normative diffusion MRI data from 178 unrelated HCP participants acquired on a ultra-high-resolution 7T MRI scanner (Siemens Medical Systems, Erlangen, Germany) were obtained from the publicly available S1200 WashU-Minn-Ox HCP dataset [25–27]; (2) diffusion, structural, and functional resting-state MRI data from 37 females recruited by the Stanford Eating Disorders Research Program acquired on a 3T MRI scanner (Discovery MR750, GE Healthcare, Milwaukee, Wisconsin); (3) diffusion and structural clinical 3T MRI data from one subject undergoing NAc DBS.

### Diffusion MRI data preprocessing and probabilistic tractography

Whereas preprocessing was performed on the diffusion MRI data from the clinical cohort and clinical trial subject to prepare the images for probabilistic tractography using the FSL suite, the normative HCP diffusion MRI data had already been preprocessed (with the minimal preprocessing pipeline) [28, 29]. The diffusion-weighted images were corrected for motion and geometric distortions using the “topup” and “eddy” functions, similar to that applied in HCP’s preprocessing pipeline. For each subject, diffusion and T1-weighted images were co-registered using boundary-based registration. Further, two successive steps of linear and nonlinear registration between the subject’s brain and the MNI 09c brain template as previously described [22, 30]. In a third step, the MNI-defined ROIs were registered to subject’s space. The NAc mask was defined on the standard MNI152 09c template adapted from CIT168 Subcortical In Vivo Probabilistic Atlas, while the vmPFC mask was defined using the Harvard-Oxford brain atlas, as used previously [31, 32]. These masks were co-registered to participant’s space using Advanced Normalization Tools (ANTs) in a previously described multi-step approach [22, 30]. FSL’s BedpostX was used to prepare the processed data for probabilistic tractography, which was performed with FSL’s Probtrackx2, using distance correction and each NAc voxel as seed and the vmPFC as target [33, 34]. A total of 5000 seed points per voxel were used to generate streamlines, and only the streamlines that reached the target were retained for further analysis. The total number of streamlines that reached the target is represented in the “waytotal”. The strength of the connections between seed and target (structural connectivity) was expressed as a tractography connectivity index (tractography-CI), as defined by Tschentscher et al. in a previous study by the formula: log(waytotal)/log(number of seed points) [35].

### Nucleus accumbens segmentation

Tractography was used to subdivide the NAc based on each individual distribution of streamlines between the NAc and vmPFC and define the NAc subregion where the vmPFC streamline are more densely located. This analysis was first performed in high-resolution, normative HCP dataset to ensure highest anatomical accuracy of NAc segmentation, and then applied to our binge-prone cohort images to compute the number of streamlines between vmPFC and NAc subregion in our condition-specific cohort. We computed the averaged streamline probability maps of NAc voxels to the vmPFC to obtain the normative weighted average group map of streamline probability between the NAc and the vmPFC across the 178 HCP subjects. Connectivity matrices with the number of streamlines between seed and target voxels were also generated for each subject. We used k-means for hypothesis-free segmentation of individual NAc subregions based on these connectivity matrices. For the case of large inter-voxel similarities in streamline count, the algorithm fails to identify two distinct clusters. Each subject’s resulting clusters were transformed to standard MNI space and concatenated into group average NAc subregions based on vmPFC interactions. Finally, we co-registered the normative clusters to our binge-prone cohort images to assess the number of streamlines (normalized by seed volume) between the vmPFC and each NAc subregion.

### Demographics, clinical and behavioral data of binge eating cohort

Participants were females recruited from September 22, 2015, to August 31, 2020. Consent was obtained according to the Declaration of Helsinki and approved by the institutional ethical committee (IRB-35204). Demographics of participants are described in Table 1. We included participants (*n* = 37) with complete clinical, behavioral, and imaging data, who engaged in binge eating, defined by at least one weekly episode of eating large amounts of food in short periods accompanied by the feeling of loss of control eating over the prior 6 months (i.e., binge-prone cohort; mean age = 26 ± 5.6 years; BMI = 27.9 ± 8.5; binge frequency = 2.7 ± 1.4 episodes/week). The number of binge eating and purging episodes per week was assessed with the Eating Disorder Examination, a standardized diagnostic interview [36]. Given the potential impact of recall bias in precising the frequency of binge eating episodes retrospectively in a diagnostic interview, we limited the usage of this variable only as a categorically defining feature of binge-proneness. Participants were also divided into two cohorts (see Table 1): (1) lean binge-prone cohort (*n* = 19): BMI < 25, at least one weekly episode of binge eating (referred to as lean cohort); (2) obese binge-prone cohort (*n* = 13): BMI > 30, at least one weekly episode of binge eating (referred to as obese cohort). Clinical and behavioral variables that differed between lean and obese cohorts were further investigated. The Beck Depression Inventory (BDI) and the Beck Anxiety Inventory were used to screen for depression and anxiety, respectively [37, 38]. The Difficulties in Emotion Regulation Scale was used to assess impairment in emotion regulation [39].

**Table 1:** Summary of demographics, behavior, and imaging measurements across within binge-prone cohort, obese binge-prone cohort, and lean binge-prone cohort.

	Binge-prone cohort				
	All *n* = 37	Lean (BMI < 25) *n* = 19	Obese (BMI > 30) *n* = 13	Mann–Whitney *U*	*p*^a^
Age	26 ± 6	26 ± 6	27 ± 6	94	0.26
BMI	28 ± 8.5	21.8 ± 1.7	37.1 ± 7.6	–	–
Binge frequency (per week)	2.7 ± 1.4	2.5 ± 1.2	2.9 ± 1.7	108	0.56
Purging frequency (per week)	0.2 ± 0.5	0.03 ± 0.1	0.36 ± 0.7	155	0.16
BDI-I	13 ± 8.1	10.3 ± 8.0	17.2 ± 6.2	62	**0.02**
BAI	10.6 ± 7.2	11.2 ± 7.6	11.2 ± 7	119	0.88
DERS	96 ± 24	89 ± 25	102 ± 23	96	0.30
vmPFC-NAc shell rsFC^b^	0.11 ± 0.19	0.16 ± 0.15	0.05 ± 0.23	149	**0.04**
vmPFC-NAc shell R CI	0.87 ± 0.03	0.87 ± 0.03	0.85 ± 0.02	191	**0.009**
vmPFC-NAc shell L CI	0.84 ± 0.04	0.86 ± 0.05	0.82 ± 0.03	195	**0.005**
vmPFC thickness R	3.36 ± 0.45	3.48 ± 0.44	3.14 ± 0.4	183	0.02
vmPFC thickness L	3.37 ± 0.49	3.46 ± 0.46	3.2 ± 0.57	162	0.15

Statistically significant *p*-values are in bold.

*BDI* Beck’s depression inventory, *BAI* Beck’s anxiety inventory, *DERS* difficulties in emotion regulation scale.

^a^Mann–Whitney *U* test comparing lean and obese unless otherwise specified.

^b^Three subjects were excluded from rsFC analysis due to imaging artifacts.

### Resting-state functional MRI data preprocessing and connectivity analysis

Resting-state fMRI scans from the binge-prone cohort were preprocessed using *fMRIPrep* 1.2.3 [40]. The preprocessing of the functional image involved skull-stripping, co-registration to the T1 reference image, and head motion and susceptibility distortion corrections. After removal of non-steady state volumes and spatial smoothing with a 6 mm FWHM isotropic Gaussian kernel, ICA-AROMA was used to identify motion-related noise components in the BOLD signal [41]. Framewise displacement (FD) and root mean square variance over voxels of the temporal derivative of time courses (DVARS) were calculated [42, 43]. Global signals were extracted within the cerebrospinal fluid (CSF), white matter (WM), gray matter, and whole-brain masks. XCP Engine 1.0 was used to perform denoising of the preprocessed BOLD output from *fMRIPrep*, utilizing the estimated confound parameters [44, 45]. This included demeaning and removal of any linear or quadratic trends and temporal filtering using a first-order Butterworth bandpass filter (0.01–0.08 Hz). These preliminary preprocessing steps were then followed by confound regression of ICA-AROMA noise components, together with mean WM, CSF, and global signal regressors. All regressors were bandpass filtered to retain the same frequency range as the data to avoid frequency-dependent mismatch [44]. Three subjects were excluded from resting-state analysis due to excessive movement as measured by (1) mean FD > 0.2 mm, (2) more than 20% of FD over 0.2 mm, or (3) any FD > 5 mm [45]. Resting-state functional connectivity analysis was performed on the binge-prone cohort’s preprocessed resting-state fMRI data using DPABI 4.3/DPARSF which is based on Statistical Parametric Mapping (SPM12, https://www.fil.ion.ucl.ac.uk/spm) [46]. A seed-based approach was performed to measure rsFC in the binge-prone cohort by calculating the rsFC between the whole vmPFC mask and the segmented mask corresponding to the NAc subregion where most tractography-defined vmPFC structural connections were localized (as described in “Results”).

### Measurement of cortical thickness

The processing of the T1-weighted image involved the use of the ANTs cortical thickness pipeline [47]. An existing template image and associated anatomical priors (https://figshare.com/articles/ANTs_ANTsR_Brain_Templates/915436) were used to run each image through the pipeline in order to generate a cortical thickness map in subject space. This processing pipeline includes bias correction, [48] brain extraction, n-tissue segmentation, and spatial normalization [49, 50]. This analysis was performed to assess thinning of vmPFC in obesity in our binge-prone cohort. ANTs was used to perform registration between the MNI template and the template used in the cortical thickness pipeline [47]. These transforms were composed with the template-to-subject transforms from the pipeline to transform the vmPFC labels into participant’s voxel-wise cortical thickness maps, to calculate mean vmPFC thickness. Of note, this analysis was conducted using public scripts from the Penn Image Computing and Science Laboratory (https://github.com/ANTsX/ANTs/tree/master/Scripts), whereas the tractography-based analysis was performed using an in-house developed script, thus slightly modified different normalization approaches were applied, both using ANTs.

### Case illustration

Informed consent was obtained prior to enrollment per the study protocol (ClinicalTrials.gov Identifier: NCT03868670) approved by the institutional ethical committee (IRB-46563) and the U.S. FDA (IDE G180079) [24]. The subject was a 56-year-old female, meeting criteria for severe obesity (BMI = 48.9 kg/m^2^) and binge-eating disorder, based on standardized eating disorder examination. She had failed multiple treatments for obesity, including pharmacological and non-pharmacological therapies as well as Roux-en-Y gastric bypass per the study protocol. The subject had no current diagnosis of mood or anxiety disorders as established by two trained psychiatrists based on standardized clinical interviews. For the duration of the study, the patient did not receive any medication implicated in weight loss. We used a preoperative high-resolution diffusion MRI protocol in one morbidly obese subject with binge-eating disorder undergoing a NAc DBS in our first-in-human clinical trial (MRI protocol details are summarized in Table S1). Episodes of uncontrolled eating were quantified in two ways: (1) a self-reported food diary in which the participant made records of these episodes right after they had occurred and (2) the Eating Loss of Control Scale (ELOCS-18) [51]. Of note, the participant-reported counts of such episodes in real-time in a food diary. This did not follow, however, the well-defined criteria described in the different items of the ELOCS-18, which relies on the participant’s recollection of eating behavior over the preceding 4 weeks. Hence, the numbers of self-reported weekly episodes of uncontrolled eating were slightly different than those formally quantified in the ELOCS-18 frequency score. Changes in body weight and BMI were secondary endpoints, given the potentially delayed nature of weight changes following changes in eating behavior. The BDI II was used to screen for depression during the study [52].

The trajectories of the electrodes were planned according to published stereotactic coordinates used to target the NAc, and then optimized with direct targeting using the Fast Gray Matter Acquisition T1 Inversion Recovery and T1 images, as well as tractography output to ensure lead placement encompassed the NAc subregion with a higher number of streamlines to the vmPFC [22, 53, 54]. Electrode location was confirmed after lead reconstruction using the thin-cut postoperative CT scan co-registered to preoperative MRI. Initially, 1 week of stimulation was initiated under blinded conditions unilaterally using the left lead only to assess safety of the intervention, as required per study protocol. Stimulation was turned off again under blinded conditions for 1 week for safety data evaluation, as required per study protocol, followed by open-label, bilateral NAc stimulation in the next 10 weeks (completing a 12-week period). Stimulation was delivered using a default continuous detector (RNS-320; NeuroPace) with settings derived from prior epilepsy experience from the literature as well as our team of neurosurgeons and interventional neuro-psychiatrists familiar with low frequency signals in electrographic recordings [55]. While preclinical studies justified this candidate biomarker approach to responsive DBS, a customized detector for binge eating has not yet been developed for humans but is subject to ongoing investigation in our active trial [11, 24].

### Statistical analysis

Statistical analyses were performed using the RStudio Version 1.2.5042 (RStudio, Inc.). Mann–Whitney *U* test was used to compare the corrected number of streamlines between the vmPFC and NAc subregions in the binge-prone cohort. Spearman’s correlation coefficients (*rho*) were computed separately between BMI and (1) vmPFC- NAc shell rsFC, (2) vmPFC-NAc shell tractography-CI, and (3) vmPFC thickness, all corrected for age, given well-described effects of aging in brain structure [56, 57]. The Spearman’s *rho* represents the strength (effect) of the correlation. Mann–Whitney *U* test was used to compare vmPFC-NAc shell rsFC, vmPFC-NAc shell tractography-CI, and vmPFC thickness between obese and lean cohort. Effect sizes of group comparisons were calculated using rank biserial correlation. The numbers of weekly episodes of uncontrolled eating during the 12-week period of active stimulation of the identified NAc subregion were compared with baseline using by the Friedman test, a non-parametric analysis of variance of related samples [58]. As described by Tomczak and Tomczak, effect size of the Friedman test was estimated as the Kendall’s *W* coefficient, which ranges from 0 (indicating no agreement) to 1 (indicating a perfect agreement) [59]. The four weeks that preceded the onset of active stimulation served as the baseline condition against which the changes in both the number of weekly episodes of uncontrolled eating and BMI were computed over the ensuing 12 weeks. Dunn’s post hoc test was used for comparisons between baseline and other weeks, with Bonferroni correction for multiple comparisons [60]. A two-tailed significance threshold (*α*) was set at 0.05 for all statistical tests.

## Results

### vmPFC-NAc streamlines converge within the NAc shell subregion

**Fig. 1:**
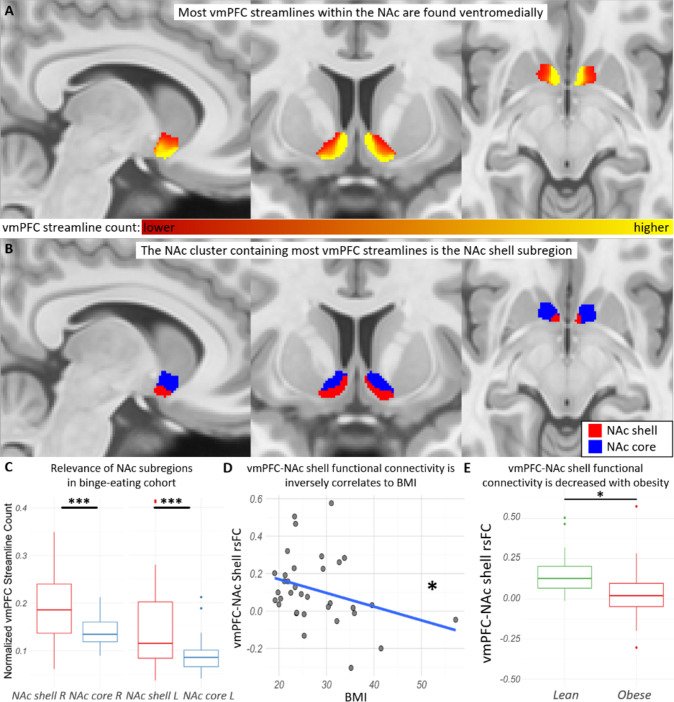
Tractography-based vmPFC streamlines converging within the NAc shell, which has lower rsFC with the vmPFC of obese cohort. **A** Group average streamline probability in normative dataset (*n* = 178) between the NAc and the vmPFC from lower (red) to higher (yellow); and **B** group average NAc shell (red) and core (blue) subregions in standard space based on the distribution of these streamlines in the normative data. Voxels present in less than 40% of the HCP subjects were excluded. **C** Significantly higher normalized streamline counts were also observed between the vmPFC and the right (*U* = 367; *p* < 0.001) and left (*U* = 363, *p* < 0.001) NAc shell as compared to the NAc core in the binge-prone cohort (*n* = 37). **D** The vmPFC-NAc shell rsFC was negatively correlated with BMI (*rho* = −0.36; *p* = 0.04) and **E** significantly lower in the obese participants (BMI ≥ 30; *U* = 149, *p* = 0.04).

We performed probabilistic tractography in a normative dataset to identify the region within the NAc where the vmPFC streamlines are more densely located (Fig. 1A). In the normative dataset, a k-means algorithm identified two subregions (masks) with distinct vmPFC connectivity patterns for all participants bilaterally (Fig. 1B). Most vmPFC streamlines converged in a ventromedial region within the NAc analogous to the previously described presumed NAc shell (defined herein as shell), while the other subregion analogous to the presumed NAc core (defined herein as core) received less vmPFC streamlines, similar to what was previously reported in rodents and primates [22, 61, 62]. To confirm relevance of this NAc segmentation in condition-specific imaging data, the NAc subregions defined in normative data were then co-registered to the imaging data of females who engaged in binge eating (*n* = 37, binge-prone cohort). The shell subregion also presented higher normalized streamline counts to the vmPFC (right hemisphere: *U* = 367, *p* < 0.001, two-tailed; left hemisphere: *U* = 363, *p* < 0.001, two-tailed) than the NAc core subregion in the binge-prone cohort (Fig. 1C).

### Decreased vmPFC-NAc shell resting-state functional connectivity with obesity

We then analyzed the available clinical and behavioral data from this binge-prone cohort (mean age = 26 ± 5.6 years; BMI = 27.9 ± 8.5; binge frequency = 2.7 ± 1.4 episodes/week). Demographics, behavior, and imaging measurements are further described in Table 1. Correlations of behavioral and imaging measurements with BMI in the binge-prone cohort are described in Table S2. Importantly, our assessments took a dimensional (RDoC-type) approach to binge-proneness, as opposed to establishing diagnosis, to investigate associations between the investigated circuit and clinical and behavioral phenotypes (e.g., binge-proneness, obesity, depression, etc.). Moreover, we limited the usage of participant’s binge frequency information only as a defining feature of binge-proneness given the potential impact of recall bias in accurately reporting the frequency of binge-eating episodes. We tested the a priori hypothesis that the NAc shell exhibited decreased rsFC with the vmPFC in the obese cohort. In line with this hypothesis, the vmPFC-NAc shell rsFC was inversely correlated with BMI in the overall binge eating cohort (*rho* = −0.36, *p* = 0.04, two-tailed) (Fig. 1D). Subjects were then divided categorically into two cohorts: (1) lean cohort (BMI < 25) and (2) obese cohort (BMI ≥ 30). The vmPFC-NAc shell rsFC was significantly lower in the obese compared to the lean cohort (*U* = 149, *p* = 0.04, two-tailed; effect size = 0.46) (Fig. 1E).

### BMI is inversely correlated with vmPFC-NAc shell structural connectivity

**Fig. 2:**
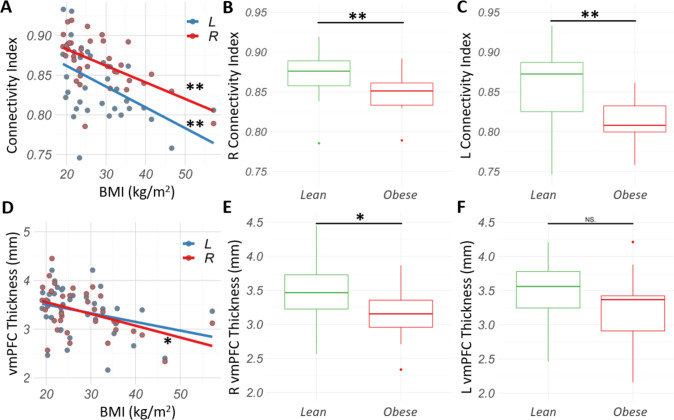
The vmPFC-NAc shell structural connectivity and vmPFC thickness are associated with the obese state. **A** BMI was inversely related to the right (*rho* = −0.50, *p* = 0.009, two-tailed) and left (*rho* = −0.50, *p* = 0.005, two-tailed) vmPFC-NAc structural connectivity indices. **B**, **C** Structural connectivity index between vmPFC and NAc was significantly decreased in right and left hemispheres structural in the obese compared to the lean cohort. **D** BMI was found to be inversely correlated to the right (*rho* = −0.42, *p* = 0.01, two-tailed) and left (*rho* = −0.29, *p* = 0.09, two-tailed) vmPFC thickness. **E** Obese cohort presented significantly lower right vmPFC thickness than the lean cohort (*U* = 183, *p* = 0.02). **F** Differences in left vmPFC thickness between obese and lean cohort did not reach statistical significance (*U* = 162, *p* = 0.15). N.S. non-significant. *p < 0.05. **p < 0.01.

BMI was found to be inversely correlated with the right (*rho* = −0.50, *p* = 0.002, two-tailed) and the left (*rho* = −0.50, *p* = 0.002, two-tailed) vmPFC-NAc tractography connectivity index (tractography-CI) (Fig. 2A). The tractography-CI is a metric of structural connectivity that controls for the volume of the seed region [35]. The vmPFC-NAc tractography-CI was found to be significantly decreased for the obese cohort in both right (*U* = 191, *p* = 0.009, two-tailed; effect size = 0.55) and left (*U* = 195, *p* = 0.005, two-tailed; effect size = 0.58) hemispheres (Fig. 2B, C).

### Decreased vmPFC thickness in the obese state

Considering our structural connectivity findings in the binge-prone cohort, we assessed whether there was a relationship between vmPFC thickness and obesity. BMI was inversely correlated with the right vmPFC thickness (*rho* = −0.42, *p* = 0.01, two-tailed) though this correlation did not reach the significance threshold on the left (*rho* = −0.29, *p* = 0.09, two-tailed) (Fig. 2D). Furthermore, the obese cohort had significantly decreased vmPFC thickness compared to the lean cohort (*U* = 183, *p* = 0.02, two-tailed; effect size = 0.48) on the right side (Fig. 2E), although decreased vmPFC thickness did not reach statistical significance on the left side (*U* = 162, *p* = 0.15, two-tailed; effect size = 0.31) (Fig. 2F).

### Depression scores do not explain vmPFC-NAc correlations with BMI

**Fig. 3:**
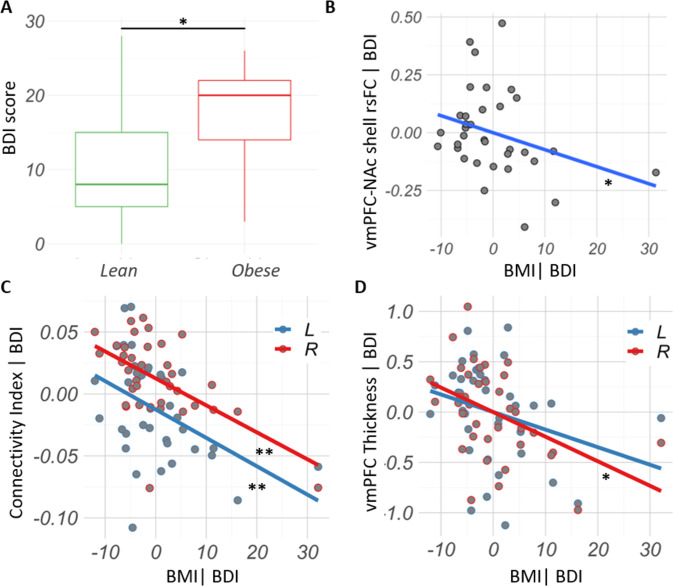
Depression scores do not explain vmPFC-NAc shell connectivity and vmPFC thickness correlations with BMI in participants with binge eating. **A** Beck’s Depression Inventory (BDI) score was increased in the obese compared to the lean cohort. **B** Correlation between BMI and vmPFC-NAc shell rsFC (*rho* = −0.36, *p* = 0.04, two-tailed) remained significant after including BDI score as a covariate (vmPFC-NAc shell rsFC | BDI). **C** Correlations between BMI and left (*rho* = −0.44, *p* = 0.008, two-tailed) and right (*rho* = −0.52, *p* = 0.002, two-tailed) vmPFC-NAc structural connectivity index remained significant after including BDI score as a covariate (Connectivity Index | BDI). **D** Correlations between BMI and right vmPFC thickness (*rho* = −0.42, *p* = 0.01, two-tailed) also remained significant after including BDI score as a covariate (vmPFC Thickness | BDI). *p < 0.05. **p < 0.01.

Depression scores (BDI) were significantly increased in the obese compared to the lean cohorts (*U* = 62, *p* = 0.02; effect size = 0.50) (Fig. 3A). Measures of binge frequency, purging behavior, anxiety and problems in emotion regulation did not differ between the lean and obese cohort (Table 1). In other words, while changes in vmPFC-NAc circuit measurements are not necessarily caused by loss of control eating, they are rather found to be associated with obesity severity in binge-prone subjects. Nonetheless, correlations between BMI and (1) vmPFC-NAc shell rsFC (*rho* = −0.36, *p* = 0.04, two-tailed) (Fig. 3B), (2) right (*rho* = −0.50, *p* = 0.002, two-tailed) and left vmPFC-NAc shell tractography-CI (*rho* = −0.44, *p* = 0.008, two-tailed) (Fig. 3C), and (3) right vmPFC thickness (Fig. 3D) (*rho* = −0.42, *p* = 0.01, two-tailed) remained significant after including depression as a covariate. BMI and left vmPFC thickness did not significantly correlate after including BDI score as a covariate (*rho* = −0.27, *p* = 0.11, two-tailed) (Fig. 3D).

### Case illustration of personalized DBS targeting of the vmPFC-NAc shell circuit

**Fig. 4:**
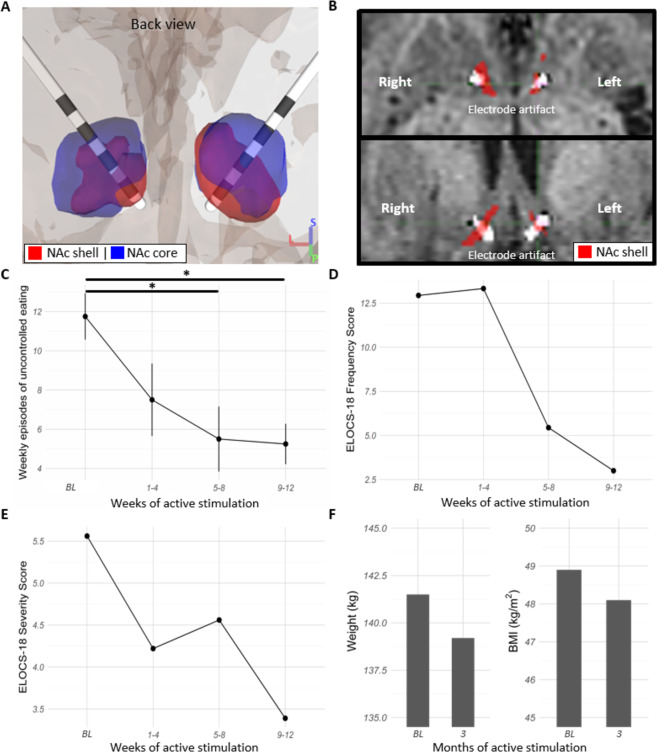
Case illustration of subject-specific NAc shell DBS. **A** Personalized NAc circuit-based subregions were defined by probabilistic tractography in high-resolution diffusion MRI and implanted with DBS electrodes in a 56-year-old female with severe obesity in our first-in-human clinical trial of NAc DBS for loss of control eating. **B** The bilateral NAc subregions with the most robust tractography-defined vmPFC structural connections (red overlay, NAc shell) were encompassed by the two distal-most electrode contact artifacts from co-registered CT scan (white overlay). **C** During 12 weeks of active stimulation, the participant sustained a statistically significant decrease in the number of weekly episodes of uncontrolled eating (Friedman’s *χ*^2^ (3) = 7.86, *p* = 0.049, two-tailed); this difference became significant in comparison to baseline levels from weeks 5–8 of active stimulation onwards (*p* < 0.05, Dunn’s test, two-tailed, FDR-corrected). **D**, **E** In the 12-week period, the participant also had a decrease in the ELOCS-18 frequency (baseline, BL: 12.9 episodes per week; weeks 9–12: 3 episodes per week) and severity (baseline: 5.6; weeks 9–12: 3.4) scores. **F** After 3 months, the participant also sustained a decreased in body weight (weight at baseline: 141.5 kg; 3-month follow-up: 139.2) and BMI (baseline: 48.9; BMI after 3-month follow-up: 48.1). *p < 0.05.

To examine a causal link between the perturbed vmPFC-NAc shell circuit and impulsive overeating in obesity, we assessed feasibility of applying probabilistic tractography to directly target the human NAc shell subregion—where vmPFC streamlines were localized—in one participant from a first-in-human clinical trial [24]. Personalized NAc subregions could be identified in the single-subject data using probabilistic tractography between the vmPFC and the NAc, as was done in the normative dataset above. The two ventral-most contacts were successfully implanted in direct contact with the tractography-defined vmPFC-NAc shell circuit-target bilaterally (Fig. 4A).

### Modulating the vmPFC-NAc shell circuit ameliorates uncontrolled eating and obesity

The participant received bilateral active stimulation of the NAc shell for 3 months in bipolar mode (0.5 mA; 80 µs; 125 Hz; charge density of 0.5–1.0 µC/cm^2^) using the two ventral-most contacts of both leads (white overlay in Fig. 4B). Guided by our preclinical studies and a previously defined protocol informed by a default approach in epilepsy, stimulation was triggered by low frequency field potential detection [10, 11, 55]. The participant began reporting feeling more in control of eating behavior and food choices within the first 24 h of this active stimulation assay. She later reported only having three meals per day since programming, without the prior usual urges to snack between meals. There was a significant overall decrease in the number of self-reported weekly episodes of uncontrolled eating (Friedman’s *χ*^2^ (3) = 7.86, *p* = 0.049, two-tailed, Kendall’s *W* = 0.655) (Fig. 4C). In comparison to baseline levels, this decrease became statistically significant in weeks 5–8 (*p* = 0.035; Dunn’s test, two-tailed, FDR-corrected), and thus remained over weeks 9–12 (*p* = 0.035; Dunn’s test, two-tailed, FDR-corrected) of stimulation. By the end of week 12, the number of self-reported weekly episodes of uncontrolled eating had dropped from a weekly average of 12 to only five. In addition to the self-reported assessments, the participant had a decrease in the ELOCS-18 frequency (baseline: 12.9 episodes per week; weeks 9–12: 3 episodes per week) and severity (baseline: 5.6 out of 10; weeks 9–12: 3.4 out of 10) scores (Fig. 4D, E). There was an associated decrease in body weight (baseline: 141.5 kg; 3-month follow-up: 139.2) and BMI (baseline: 48.9; 3-month follow-up: 48.1) following only 3 months of NAc shell stimulation (Fig. 4F). No change in BDI scores was observed during the 12-week period (baseline score: 3; weeks 9–12: 3).

## Discussion

Disinhibited eating modeled in our analysis using binge-eating behavior is a strong predictor of treatment-refractoriness in obesity, leading to worse outcomes even to the most aggressive treatments [3, 9]. Unraveling neural correlates of both disinhibited eating and the obese state could provide unique insights into mechanisms of treatment-refractoriness and guide development of neuromodulatory therapies. To our knowledge, this is the first study to (1) provide a vmPFC-NAc circuit-based segmentation of a human NAc subregion, the NAc shell, where vmPFC-NAc structural connections were located, (2) find condition-specific, functional, and structural effects of BMI on the vmPFC-NAc shell circuit, and (3) demonstrate feasibility of directly targeting the vmPFC-NAc shell circuit with DBS in a human subject as part of a clinical study aimed at improving eating behavior and associated obesity.

We had previously found using diffusion MRI data from the HCP as well as a single case study that probabilistic tractography can be utilized to subdivide the human NAc into core and shell subregions based on whole-brain connectivity profiles [22]. This methodology has been applied successfully to other brain regions as well [30]. We have now confirmed, in both normative and single-subject diffusion MRI data, that two NAc subregions can be differentiated using probabilistic tractography based on connectivity to the vmPFC alone; with the NAc shell concentrating most vmPFC streamlines. The transferability of this finding from the normative dataset was confirmed by comparing probabilistic tractography between the vmPFC and the tractography-defined NAc shell and core, separately, in the binge-prone cohort. In this dataset, acquired using standard imaging parameters, we also observed a markedly larger number of vmPFC streamlines in the NAc shell compared to the NAc core (corrected for volume). This is in line with previous findings using 3D histology-based tractography and synaptic tracing studies in rodents and primates [21, 22, 62, 63].

More than a decade of functional neuroimaging studies carried out in adult volunteers have revealed associations between overall prefrontal activity and BMI [64, 65]. While many other studies addressed structural and functional brain changes in subjects with binge-related disorders, a recent systematic review revealed a lack of studies investigating brain circuits underlying the obese state in binge-prone individuals [66, 67]. The functional relevance of the NAc shell subregion with a higher number of vmPFC streamlines is supported by negative correlation between BMI and rsFC of this vmPFC-NAc shell circuit. Moreover, decreased rsFC within this circuit in the obese cohort further supports disease-specific alterations. While prior work had found greater vmPFC-NAc connectivity in overweight and obese women in a specific fMRI frequency band, the vmPFC cluster that was used was more rostral, in the frontopolar cortex, as opposed to the definition of the vmPFC used here which includes the subgenual cortex; moreover, that study excluded subjects with disordered eating [68].

Widespread white matter microstructure alterations have been reported in subjects with binge eating [69]; however, white matter alterations specific to binge-prone individuals with obesity have not been explored. The present investigation is the first to provide evidence supporting decreased structural connectivity between the NAc shell and vmPFC in females with comorbid obesity and binge eating compared to lean females with binge eating alone. This is in line with recent work using positron emission tomography that revealed lower synaptic density in subjects with obesity and psychiatric comorbidities [70]. More specifically, our finding likely represents that the vmPFC-NAc shell is perturbed in subjects with obesity and binge eating. Furthermore, the finding that BMI was inversely related to the vmPFC-NAc shell tractography-CI supports an aberration in this brain circuit in the obese cohort and perhaps even a vulnerability to obesity [19, 71]. This is in line with previous empirical imaging assays and computational models that found robust relationship between tractography and rsFC where structural connections are present [72]. We also found that cortical thickness within the vmPFC region is decreased in the obese cohort, and inversely related to BMI. Perhaps not surprisingly, vmPFC-NAc shell circuit perturbation is accompanied by thinning of the vmPFC in the obese cohort, what may represent a predisposition for obesity in some subjects. Another prior study that focused on the association between thickness of frontal cortices and BMI found that the cortical thickness of the right superior frontal gyrus was a predictor of BMI [73]. Unfortunately, vmPFC thickness was not included in the regression model and eating disinhibition scores were unrelated to the measures of cortical thickness that were included. Given the vmPFC role in inhibitory control of reward-seeking behavior, decreased vmPFC thickness in the obese state may render this inhibitory system unable to prevent binge eating [18, 19].

Clinical measures of depression, anxiety and emotion regulation have been reported to be impaired in obese subjects with a history of binge eating[6, 8]. When comparing the obese cohort with the lean cohort using a psychiatric battery of scales, the only variable that we found to differ between these cohort was the BDI score. Moreover, the vmPFC-NAc circuit has repeatedly been implicated in mediating mood states [17, 74, 75]. Nevertheless, this measure of depression did not fully explain the key finding that BMI negatively impacts structural connectivity of the vmPFC-NAc circuit.

Importantly, it should not be inferred from these results that this impulse control circuit is the only (or even the most) meaningful circuit associated with obesity in subjects prone to binge eating. This work adds instead to the large body of literature implicating the vmPFC-NAc circuit in impulse control. This study does not attempt to examine effects of the obese state on other circuits as it is expected that there are many circuit-related correlates. Moreover, enrollment in this study was negatively impacted by the COVID-19 pandemic and thus implications from our study with binge-prone females are limited by sample size. There is also a lack of participants without binge eating; thus, we could not determine whether findings in an obese cohort not prone to binge eating would differ from our obese cohort. One systematic review has found the impulse control circuit to be significantly impaired in obese humans regardless of the presence of a formal diagnosis of binge eating disorder [2]. Furthermore, to ensure vmPFC structural connectivity results were not moderated by sex, as found in previous studies, our study only included females [76]. Notwithstanding, this precluded sex differences assessment and may raise the question as to whether our findings would apply to males. In line with the potential generalizability of our findings, one study also found thinning of the vmPFC to be related to BMI in a large sample of males and females volunteers [77]. That study, however, did not include any information related to binge eating or perform circuit-based analyses.

We have also demonstrated the feasibility of the immediate clinical application of this novel targeting methodology using preoperative diffusion MRI. The feasibility of circuit-based targeting was confirmed by evaluating lead placement in postoperative imaging. In fact, modulating this vmPFC-NAc circuit with DBS exhibited a direct relationship to the subject’s disinhibited eating given restoration in self-control (i.e., decreased frequency of uncontrolled eating) followed by a decrease in BMI. These encouraging findings in a rare case should be interpreted with caution in terms of its applicability to larger population, as this participant was enrolled in a first-in-human, early feasibility study, which requires following very strict criteria (e.g., lack of psychiatric comorbidities) that may not reflect the majority of the population of individuals with obesity. This participant was blinded to the timing of DBS onset. Furthermore, in the described study participant, the decrease in frequency of uncontrolled eating episodes is evidence for a potential effect of NAc DBS in re-establishing the function of the targeted impulse control circuit [14]. While we cannot fully establish the causal relationship between changes in eating behavior and stimulation of this specific circuit in a single case illustration, prior mouse studies revealed increased calcium activity in response to intermittent DBS of the NAc shell, which may explain a possible rescue effect on the function of this perturbed circuit. Also, although the improvement in eating-related impulse control was accompanied by preliminary weight loss after 3 months of intervention in our case illustration, long-term follow-up may be necessary to evaluate whether this could amount to a clinically meaningful change over time. Notably, the case illustration described in this manuscript leverages an ongoing early feasibility study using closed-loop DBS. Given the complexities and intricacies of the neural recordings and the stimulation triggered by these neural signatures, further work will be focused in describing this investigational treatment in detail, which would be outside the scope of the methods described here.

In conclusion, our findings indicate that the vmPFC-NAc shell circuit is perturbed in obesity and modulating these circuit interactions within the NAc shell may ameliorate disinhibited eating. These findings pave the path forward for the application of this personalized, circuit-based targeting method to optimize neuromodulatory treatments for subjects with refractory obesity and binge eating.

## Supplementary information

Supplementary Materials

## Data Availability

Deidentified data that support the findings of this study as well as the scripts used for analyses are available from the corresponding author upon reasonable request.
