# Supplementary material for: Aberrant impulse control circuitry in obesity
Source: Mol Psychiatry. 2022 Jun 14;27(8):3374–84. doi: 10.1038/s41380-022-01640-5 (PMC9192250; doi:10.1038/s41380-022-01640-5)
Supplement: Supplementary file 1 — Supplementary Materials [file 41380_2022_1640_MOESM1_ESM.pdf]

Supplementary Materials

Supplementary Table 1. Summary of MRI acquisition parameters.

	HCP Dataset	Recruited Participants			Clinical Trial Subject	
**Data Type**	Diffusion	Diffusion	Structural (T1w)	Resting-state	High-resolution Diffusion	Structural (T1w)
**Magnetic Field**	7T	3T	3T	3T	3T	3T
**Sequence**	Spin-Echo EPI	Spin-Echo EPI	BRAVO	SPIRAL	EPI	BRAVO
**TE / TR (ms)**	71.2 / 7000	97.5 / 12800	1.87 / 5.8	30 / 2000	87* / 12235*	3.2 / 8.2
**Flip Angle (°)**	90	90	11	80	90	13
**Field-of-View (mm²)**	210	220	230	220	225	220
**Matrix Size**	200 (acquisition) x 200 (recon)	128 (acquisition) x 256 (recon)	192 (acquisition) x 256 (recon)	128 (acquisition) x 64 (recon)	150 (acquisition) x 256 (recon)	256 (acquisition) x 512 (recon)
**In-plane Res. (mm²)**	1.05x1.05	0.8594x0.8594	0.9375x0.9375	3.4x3.4	0.88x0.88	0.5x0.5
**Slice Thickness (mm)**	1.05	2.3	1.2	3.5	1.5	1
**Number of Slices**	132 Axial	74 Axial	146 Axial	31 Axial	80 Axial	176 Axial
**Runs**	4	1	1	1	1	1
**Acquisition Time (min)**	9:50 (39:20 total)	15:23	8:40	8:00	27:32*	3:42
**Diffusion Encoding (s/mm²)**	6 × b=0 65 dir. × b=1000, 65 dir. × b=2000	1 × b=0 37 dir. × b=1000	N/A	N/A	6 × b=0 128 dir. × b=1500	N/A

* For the clinical trial subject, images were acquired in a large-bore clinical scanner.

Supplementary Table 2. Correlations of behavioral and imaging measurements with BMI in binge-prone cohorts.

	**Binge-prone cohort (n = 37)**	
	Spearman's rho	*p**
Binge frequency x BMI	.04	.81
BDI-I x BMI	.34	**.04**
BAI x BMI	-.01	.94
DERS x BMI	.21	.22
vmPFC-NAc shell rsFC x BMI	-.36	**.04**
vmPFC-NAc shell R CI x BMI	-.50	**.002**
vmPFC-NAc shell L CI x BMI	-.50	**.002**
vmPFC thickness R x BMI	-.42	**.01**
vmPFC thickness L x BMI	-.29	.09

BDI = Beck's depression inventory; BAI = Beck's anxiety inventory; DERS = difficulties in emotion regulation scale.
